# Multimodality imaging assessment of caseous mitral annular calcification in a hemodialysis patient undergoing atrial fibrillation ablation: A case report

**DOI:** 10.1016/j.radcr.2026.04.080

**Published:** 2026-05-28

**Authors:** Jingyan Niu, Wencai Huang, Jiani Zou

**Affiliations:** a Department of Radiology, Medical College of Wuhan University of Science and Technology, Wuhan, Hubei 430065, China; b Department of Radiology, General Hospital of Central Theater Command of People’s Liberation Army, Wuhan, Hubei 430060, China

**Keywords:** Mitral annular calcification, Caseous mitral annular calcification, Atrial fibrillation ablation, Multimodality cardiac imaging, Cardiac computed tomography angiography, Hemodialysis

## Abstract

Caseous mitral annular calcification (CMAC) is a rare morphological variant of mitral annular calcification. On echocardiography, it often appears as a mass-like echogenic lesion and may be easily misdiagnosed as an intracardiac thrombus or tumor, posing a diagnostic challenge in clinical practice. The clinical significance and potential procedural implications of CMAC in patients undergoing catheter ablation for atrial fibrillation remain poorly understood, and relevant reports are scarce. Here, we report a patient with end-stage renal disease on long-term hemodialysis who was found to have CMAC and successfully underwent radiofrequency catheter ablation for atrial fibrillation, with favorable postoperative recovery. This case highlights the critical role of multimodality imaging in the accurate diagnosis and clinical decision-making for CMAC.

## Case summary

A 51-year-old woman was admitted with a 5-year history of recurrent palpitations and intermittent chest discomfort, which had worsened over the preceding month. The episodes were paroxysmal, lasting from several seconds to minutes and resolving spontaneously, occasionally occurring after hemodialysis sessions. She denied syncope, fever, chest pain, or any history of thromboembolic events. On physical examination, vital signs were stable, and no pathological cardiac murmurs were detected. Her medical history was notable for renal transplantation in 2006, followed by graft failure due to chronic rejection, and she was subsequently diagnosed with end-stage renal disease (ESRD). Since 2013, she had been receiving maintenance hemodialysis three times per week. She also had a history of hypertension, renal anemia, renal osteodystrophy, secondary hyperparathyroidism, and hyperphosphatemia. In 2019, she was first diagnosed with paroxysmal atrial fibrillation (AF) and had been treated with long-term anticoagulation and rate-control therapy. However, her palpitations gradually worsened and significantly impaired her quality of life, leading to the diagnosis of symptomatic, drug-refractory AF.

**Fig. 1:**
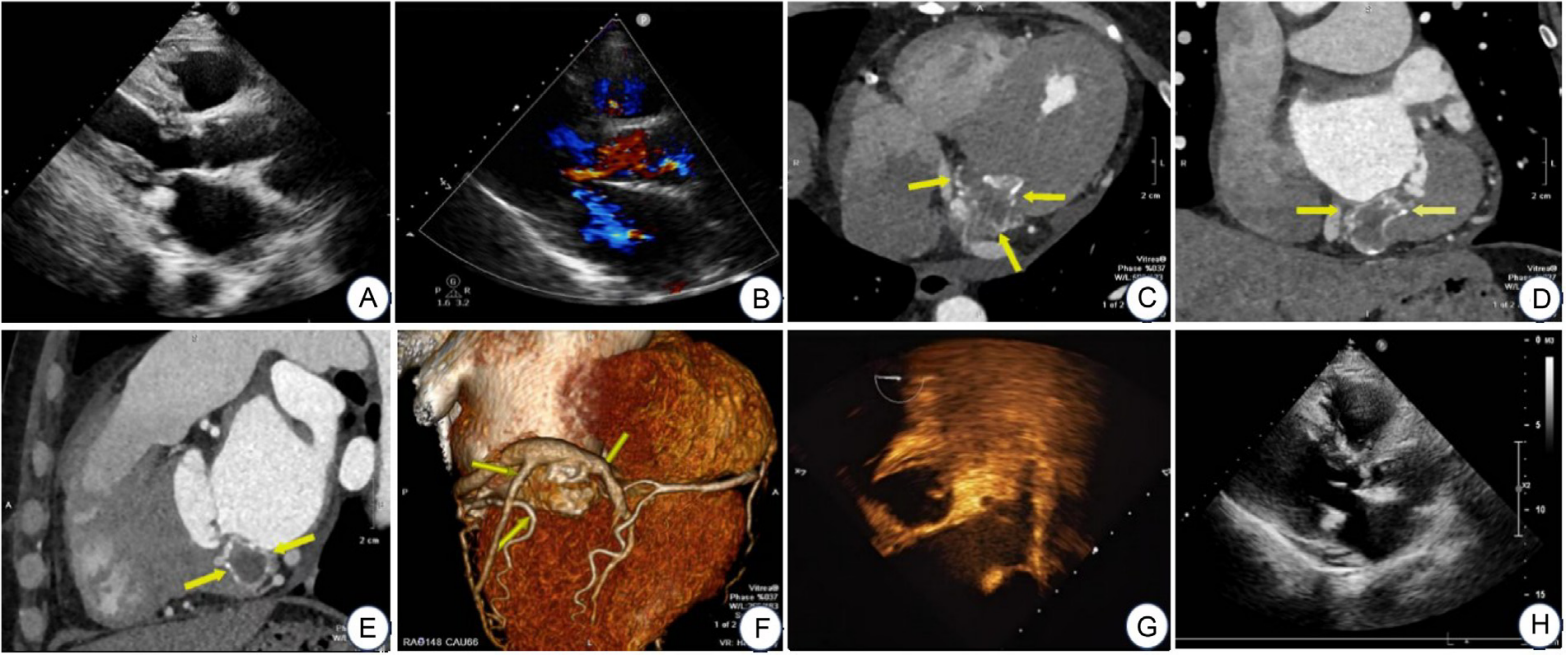
Fig. (A–B) Transthoracic echocardiography (TTE). Fig. A (parasternal long-axis view) shows a well-defined hyperechoic mass along the posterior mitral annulus. Fig. B (color Doppler) demonstrates no internal blood flow, suggesting a nonvascular lesion. Fig. (C–F) Coronary computed tomography angiography (CCTA). Figs. C–E (axial, coronal, and sagittal MIP images) demonstrate a ring-shaped heterogeneous lesion along the posterior mitral annulus (yellow arrows) with eggshell-like calcification, characterized by a hyperdense rim and hypodense center, clearly separated from the left atrial and ventricular cavities. Fig. F (VR image) shows circumferential mitral annular calcification and its spatial relationship with the coronary arteries. Fig. (G–H) Transesophageal echocardiography (TEE). No spontaneous echo contrast or thrombus is detected in the left atrium or left atrial appendage.

Electrocardiography showed sinus rhythm with nonspecific ST-T abnormalities. Laboratory findings were consistent with chronic renal insufficiency-related abnormalities. Serial transthoracic echocardiography demonstrated biatrial enlargement, with left atrial dimensions of approximately 45-46 mm (anteroposterior), 46-47 mm (transverse), and 63 mm (superoinferior), whereas the left ventricle was not enlarged. Multiple echogenic foci were noted along the posterior mitral annulus, with the most prominent focus measuring approximately 14 mm. Color Doppler showed moderate mitral regurgitation without evidence of significant mitral stenosis, and no detectable intralesional blood flow signal. Left ventricular systolic function was preserved, with FS 36.9%-39.2%, EF 67%-70%, SV 65.3-81.4 mL, and CO 5.35-5.62 L/min. Coronary computed tomography angiography (CCTA) demonstrated the full peri-annular extent of the lesion, measuring approximately 32.2 mm x 33.2 mm x 24.8 mm. The lesion was characterized by a densely calcified peripheral rim and a relatively hypodense central component, without evidence of invasive growth or abnormal contrast enhancement. These findings were consistent with caseous mitral annular calcification (CMAC). A small pericardial effusion was also noted. Transesophageal echocardiography showed no spontaneous echo contrast or thrombus in the left atrium or left atrial appendage. Dedicated quantitative indices of left atrial mechanical function were not routinely acquired. Based on the patient's clinical symptoms, medical history, and multimodality imaging findings, the diagnoses of symptomatic drug-refractory paroxysmal AF, CMAC, and ESRD were established. The patient was therefore scheduled to undergo catheter radiofrequency ablation for AF. After exclusion of procedural contraindications, radiofrequency catheter ablation was successfully performed. No perioperative complications, including bleeding, embolic events, or infection, occurred. During postoperative follow-up, the patient reported marked improvement in palpitations and a favorable clinical recovery (Figs. 1A–H).

## Discussion

Mitral annular calcification (MAC) is increasingly recognized as a complex metabolic cardiovascular disorder rather than a purely age-related degenerative process [1]. Caseous mitral annular calcification (CCMA), a rare MAC subtype, accounts for only 0.64% of MAC cases, with a prevalence of approximately 0.068% in the general population [2]. Patients with end-stage renal disease (ESRD) undergoing long-term hemodialysis are particularly susceptible to CCMA due to chronic disturbances in calcium–phosphate metabolism, secondary hyperparathyroidism, and persistent systemic inflammation, all of which accelerate cardiovascular calcification [3]. Pathologically, CCMA is characterized by a dense calcified shell surrounding a central liquefied, caseous necrotic core composed of calcium deposits, fatty acids, cholesterol, and phospholipid-rich degradation products derived from inflammatory cells. This pathological substrate is thought to result from macrophage-mediated degeneration and necrosis within advanced MAC [4]. Although the patient in the present case was relatively young, the long history of hemodialysis strongly supports a metabolism- and inflammation-driven mechanism underlying the development of CCMA.

The major diagnostic challenge of CCMA lies in its pseudotumoral appearance. The characteristic pathological structure explains its mass-like imaging features and frequent misdiagnosis as peri-annular tumors, myocardial abscesses, or atrioventricular groove abnormalities [5]. Transthoracic echocardiography remains the initial screening modality but lacks specificity. Contrast echocardiography may be helpful; however, the densely calcified rim of CCMA can generate acoustic artifacts that mimic contrast enhancement, resulting in false-positive interpretations. In contrast, cardiac computed tomography angiography (CTA) reliably demonstrates the hallmark features of CCMA, including peripheral dense calcification and a centrally hypoattenuating core, enables quantitative attenuation analysis suggestive of liquefied or lipid-rich content, and confirms the absence of true enhancement [6]. In the present case, CTA was crucial for defining the lesion’s anatomical origin and tissue characteristics, thereby excluding tumor or thrombus.

Beyond diagnostic considerations, CCMA has important procedural implications in patients undergoing atrial fibrillation (AF) ablation. Transesophageal echocardiography (TEE) is the gold standard for excluding left atrial and left atrial appendage thrombus prior to AF ablation [7]. However, CCMA located along the posterior mitral annulus may appear on TEE as a highly echogenic mass closely adjacent to the left atrial wall. When its anatomical location and pathological nature are not fully appreciated, such a lesion may be misinterpreted as a mural thrombus. Given the presence of a liquefied, caseous core, this misinterpretation can have significant clinical consequences, potentially leading to unnecessary postponement or cancellation of ablation procedures, or inappropriate intensification of anticoagulation therapy [8]. The present case illustrates that integration of CTA with TEE enables accurate localization and characterization of the lesion, confirming its structural, non-thrombotic nature and thereby facilitating informed procedural decision-making.

From a therapeutic standpoint, CCMA generally follows a benign clinical course, and conservative management is recommended in most cases. Surgical intervention is typically reserved for patients with severe mitral valve dysfunction, embolic events, or lesions in which malignancy cannot be definitively excluded [9]. Given the pathological composition of CCMA, rupture or extrusion of the caseous material has been reported in rare circumstances, underscoring the need for caution during catheter-based procedures. In patients without hemodynamic compromise or prior embolic events, isolated CCMA does not constitute an indication for surgery and should not be regarded as an absolute contraindication to AF ablation. Nevertheless, the use of three-dimensional electroanatomical mapping or intracardiac echocardiography (ICE) is advisable to ensure precise catheter navigation, avoid mechanical disruption of the calcified capsule, and minimize the theoretical risk of embolization [10].

In summary, although caseous mitral annular calcification is uncommon among long-term hemodialysis patients, its distinctive pathological structure underlies its characteristic imaging features and clinically relevant diagnostic pitfalls. In the setting of atrial fibrillation ablation, a comprehensive multimodality imaging approach—particularly incorporating cardiac CTA—is essential for accurate diagnosis, avoidance of misinterpretation, and safe procedural planning.

## Patient consent

Written informed consent for publication of this case report and associated images was obtained from the patient.
